# Detection of mild cognitive impairment in Parkinson’s disease using gradient boosting decision tree models based on multilevel DTI indices

**DOI:** 10.1186/s12967-023-04158-8

**Published:** 2023-05-08

**Authors:** Boyu Chen, Ming Xu, Hongmei Yu, Jiachuan He, Yingmei Li, Dandan Song, Guo Guang Fan

**Affiliations:** 1 Department of Radiology, The First Hospital of China Medical University, No. 155 Nanjing North Street, Shenyang, 110001 Liaoning China; 2 Shenyang University of Technology, No.111, Shenliao West Road, Shenyang, 110870 Liaoning China; 3 Department of Neurology, The First Hospital of China Medical University, No. 155, Nanjing North Street, Shenyang, 110001 Liaoning China

**Keywords:** Mild cognitive impairment, Parkinson’s disease, Magnetic resonance imaging, Diffusion tensor imaging, Machine learning

## Abstract

**Background:**

Cognitive dysfunction is the most common non-motor symptom in Parkinson’s disease (PD), and timely detection of a slight cognitive decline is crucial for early treatment and prevention of dementia. This study aimed to build a machine learning model based on intra- and/or intervoxel metrics extracted from diffusion tensor imaging (DTI) to automatically classify PD patients without dementia into mild cognitive impairment (PD-MCI) and normal cognition (PD-NC) groups.

**Methods:**

We enrolled PD patients without dementia (52 PD-NC and 68 PD-MCI subtypes) who were assigned to the training and test datasets in an 8:2 ratio. Four intravoxel metrics, including fractional anisotropy (FA), mean diffusivity (MD), axial diffusivity (AD), and radial diffusivity (RD), and two novel intervoxel metrics, local diffusion homogeneity (LDH) using Spearman’s rank correlation coefficient (LDHs) and Kendall’s coefficient concordance (LDHk), were extracted from the DTI data. Decision tree, random forest, and eXtreme gradient boosting (XGBoost) models based on individual and combined indices were built for classification, and model performance was assessed and compared via the area under the receiver operating characteristic curve (AUC). Finally, feature importance was evaluated using SHapley Additive exPlanation (SHAP) values.

**Results:**

The XGBoost model based on a combination of the intra- and intervoxel indices achieved the best classification performance, with an accuracy of 91.67%, sensitivity of 92.86%, and AUC of 0.94 in the test dataset. SHAP analysis showed that the LDH of the brainstem and MD of the right cingulum (hippocampus) were important features.

**Conclusions:**

More comprehensive information on white matter changes can be obtained by combining intra- and intervoxel DTI indices, improving classification accuracy. Furthermore, machine learning methods based on DTI indices can be used as alternatives for the automatic identification of PD-MCI at the individual level.

**Supplementary Information:**

The online version contains supplementary material available at 10.1186/s12967-023-04158-8.

## Background

Parkinson’s disease (PD) is the second most common neurodegenerative disease. Cognitive dysfunction is one of the most common nonmotor symptoms of PD [1], including PD with mild cognitive impairment (PD-MCI) and PD with dementia (PDD). PD-MCI is an intermediate state between PD with normal cognition (PD-NC) and PDD, with a prevalence of approximately 30% [2], that can progress over time to either PD-NC or PDD [1, 3]. However, cognitive decline tends to be slow and insidious, and PD-MCI is often overlooked by patients and clinicians. Once the disease progresses to PDD, it seriously affects the quality of life of the patient. Accurate diagnosis of PD-MCI is essential for effective intervention and prevention of PDD.

The current diagnosis of PD-MCI mainly depends on clinical symptoms and neuropsychological tests, but some challenges remain in terms of the homogeneity of the neuropsychological test results, and the testing process is time consuming and labour intensive. Therefore, there is a need for an easier and more accurate method to establish the diagnosis of PD-MCI.

The classical mechanism underlying PD-MCI is the abnormal accumulation of Lewy bodies [4] and β-amyloid (Aβ) [5] in neuronal cell bodies and axons accompanied by damage to glial cells, demyelination of axons, and increased microglial concentrations in the extracellular space. Structural MR studies [6–8] have confirmed that the progression of cognitive impairment in PD is closely related to white matter (WM) damage and that the range of WM hyperintensity is a moderate risk factor for cognitive impairment [7]. Moreover, WM microstructural changes occur prior to grey matter volume atrophy [9].

Diffusion tensor imaging (DTI) techniques are currently recognized as the most reliable noninvasive methods for quantifying WM fibre integrity, demonstrating greater sensitivity than conventional MRI in revealing WM microstructural damage [10]. Previous DTI studies calculated a series of intravoxel DTI indices, including fractional anisotropy (FA), mean diffusivity (MD), axial diffusivity (AD), and radial diffusivity (RD), based on a diffusion tensor model and confirmed that the WM microstructure deteriorates across stages of cognitive decline in PD patients [8]. Specifically, decreased FA and increased MD, mainly in the bilateral frontal white matter [9, 11], corpus callosum [12], and temporal regions [13], are related to cognitive dysfunction. Recently, Gong et al. proposed a novel intervoxel metric named local diffusion homogeneity (LDH) [14]. LDH is independent of the diffusion model and can reveal intervoxel diffusion properties by capturing the overall coherence of water molecule diffusion within a neighbourhood; it reflects the microstructural coherence of the underlying WM fibres and provides additional insights beyond traditional intravoxel metrics. Some recent studies have applied LDH parameters to the detection of WM microstructure abnormalities in vascular cognitive impairment [15], epilepsy [16], type 2 diabetes [17], and blepharospasm [18], demonstrating regions of variation that differed from those of intravoxel diffusion metrics. Moreover, LDH can help predict the prognosis of stroke patients [19]. However, LDH alterations in PD or PD-MCI patients have not been fully explored.

In summary, traditional statistical methods for comparing groups have demonstrated significant differences in WM microstructure between PD-MCI patients and PD-NC patients, providing new evidence for understanding the pathophysiological mechanisms underlying cognitive dysfunction in PD. However, these studies have not been translated into suitable biomarkers for identifying PD-MCI at the individual level. Additionally, it is unknown which metric is the most accurate and useful for predicting PD-MCI. In particular, the role of LDH is unclear. Machine learning classification provides a powerful method for predicting an individual’s disease status based on MRI data and has been applied to generate imaging biomarkers for various neurodegenerative diseases, such as Alzheimer’s disease [20] and Parkinson’s disease [21]. Tree model algorithms, such as decision tree (DT), random forest (RF), and eXtreme gradient boosting (XGBoost), are relatively basic and widely used classes of models in machine learning. These tree models are built with a small amount of data, have a moderately complex algorithm time, and are more interpretable than neural network algorithms. Studies have confirmed the potential of tree model algorithms in studies on automatic PD identification [21].

This study aimed to develop a machine learning model based on DTI data to automatically classify PD patients without dementia as PD-MCI and PD-NC, thus providing a more convenient method for the early clinical detection of MCI. We hypothesized that tree models employing the means of DTI indices extracted from atlas-based WM segmentation as input features would be helpful for PD-MCI diagnosis, and combining intra- and intervoxel DTI metrics could improve prediction precision. Finally, we assessed the correlations between the regional DTI parameter values of selected features and neuropsychological scores and calculated the importance of the features of the best model using the SHapley Additive exPlanation (SHAP) method to validate and explain the model.

## Materials and methods

### Participants and ethics

A total of 133 PD patients were recruited from the Department of Neurology of the First Hospital of China Medical University from June 2013 to June 2019. All subjects were right-handed and had no contraindications for MR. The inclusion criteria were as follows: (1) the PD clinical diagnostic criteria of the Movement Disorder Society (MDS) were met; (2) age older than 45 years; and (3) Hoehn and Yahr stage < 5. The exclusion criteria were as follows: (1) Parkinson’s dementia [22]; (2) severe heart, liver, kidney, or endocrine system diseases; (3) severe mental illness; (4) inability to cooperate with the MRI examination and clinical assessment; and (5) unusual structural MR findings. MR scans and clinical symptom assessments were conducted on patients in the “off” state (i.e., discontinued antiparkinsonian medications for at least 12 h). Additionally, 100 sex-, age-, and education year-matched healthy people without neurological or mental diseases were included as the healthy control group.

This study was approved by the Ethics Committee of the First Hospital of China Medical University, and all subjects gave informed consent prior to participation.

### Clinical evaluation

Each subject underwent a battery of neuropsychological tests. Motor symptom severity was measured by the MDS revision of the Unified Parkinson’s Disease Rating Scale (MDS-UPDRS) [23] Part III. Disease staging was performed using Hoehn and Yahr (H&Y) staging. The Mini-Mental State Examination (MMSE) and Montreal Cognitive Assessment (MoCA) were used to assess the patients’ global cognitive status, and the Hamilton depression scale (HAMD) was used to assess patients’ level of depression. The levodopa equivalent daily dose (LEDD) was used to summarize the patients’ medication received. In addition, the Auditory Verbal Learning Test (AVLT), Clock Drawing Test (CDT), and Trail Making Test A and B (TMT-A, TMT-B) were used to evaluate patients’ verbal memory function, visuospatial function, and executive function, respectively.

### Diagnosis of PD-MCI and PD-NC

PD-MCI was diagnosed according to the MDS Task Force level 1 criteria [2, 24], which entailed MoCA scores < 26 [25] or at least two neuropsychological test scores 2 standard deviations (SD) below the healthy control group mean and reports from the patient or family members of subjective cognitive decline, defined by a score of ≥ 1 on item 1 (cognitive impairment) of the MDS-UPDRS Part I. Participants who did not qualify for the above criteria were defined as PD patients with normal cognition (PD-NC).

### DTI data acquisition and preprocessing

A Magnetom Verio 3.0 T MRI scanner (Siemens Medical Solutions, Erlangen, Germany) equipped with a 32-channel head coil was used to obtain MRI scans of all subjects. The scanning parameters were as follows: repetition time (TR)/echo time (TE) = 10,300/95 ms, field of view (FOV) = 256 × 256 mm^2^, matrix = 128 × 128, voxel size = 2.0 × 2.0 × 2.0 mm^3^, slice thickness = 2 mm, number of directions = 64, *b* = 1000 s/mm^2^. DTI data preprocessing and atlas-based analysis (ABA) were carried out with FSL 5.0.9 (https://fsl.fmrib.ox.ac.uk/) and PANDA (Pipeline for Analysing braiN Diffusion imAges, http://www.nitrc.org/projects/panda/). The preprocessing steps included format conversion, mask generation and cropping, head motion and eddy correction, and spatial registration. Details for data acquisition and preprocessing are presented in Additional file 1.

### Feature extraction

In this study, we calculated six different DTI indicators. Four commonly evaluated intravoxel diffusivity metrics, FA (a normalized SD of the eigenvalues), MD (a direction-averaged measure), AD (apparent diffusivity parallel to the underlying tissue tract), and RD (apparent diffusivity perpendicular to the underlying tissue tract), were obtained from the tensor matrix. In addition, we calculated an intervoxel diffusivity metric called local diffusion homogeneity (LDH) using Spearman’s rank correlation coefficient (LDH) and Kendall’s coefficient concordance (LDHk); the specific calculations were performed according to a previous study [14]. The atlas-based analysis (ABA) method in the PANDA software package was selected for feature extraction. According to the John Hopkins University ICBM-DTI-81 White Matter Labels and John Hopkins University White Matter Tractography (http://cmrm.med.jhmi.edu) atlases [26], the whole-brain WM was divided into 70 regions of interest, and the mean DTI parameters were extracted for each region. Ultimately, 280 intravoxel [(FA, MD, AD, RD) *0 regions] and 140 intervoxel [(LDHs, LDHk) *70 regions] indices were extracted for each subject.

### Feature selection

First, we randomly divided the data into training and test datasets (80%:20%); the ratio of PD-MCI to PD-NC remained unchanged in this division. The training dataset was used for feature selection and model construction, and the test dataset was used to evaluate the performance of the model. A feature selection procedure was performed to remove redundant features to prevent model overfitting. First, all features were normalized by L2 normalization. Next, the random forest (RF) feature selection algorithm was applied to rank the importance of each feature, 10-fold cross-validation was performed, and the top 3% most important features were retained. Finally, Pearson correlation coefficient was used to analyse the correlations among the remaining connectome features. When the absolute value of the correlation coefficient was ≥ 0.7 and the p value was < 0.05, the feature with the lower importance was excluded. For separate intravoxel/intervoxel metrics and combined metrics, feature selection was performed as described above to construct the optimal subset of features.

### Model construction, evaluation and interpretation

We selected decision tree (DT), random forest (RF), and extreme gradient boosting (XGBoost) as the machine learning algorithms to build classifiers to distinguish PD-MCI from PD-NC. The hyperparameters were tuned with the gradient descent method and are shown in Additional file 4: Table S1.

The predictive performance of each model and the receiver operating characteristic (ROC) curve were plotted, and the area under the curve (AUC), accuracy, sensitivity, specificity, positive predictive value (PPV) and negative predictive value (NPV) were calculated. To compare the performance among different models, the DeLong test was used to compare the differences among different AUCs, and *p* < 0.05 (two-tailed) was considered statistically significant. Afterwards, the values of each selected feature between the two groups were also calculated and compared.

**Fig. 1:**
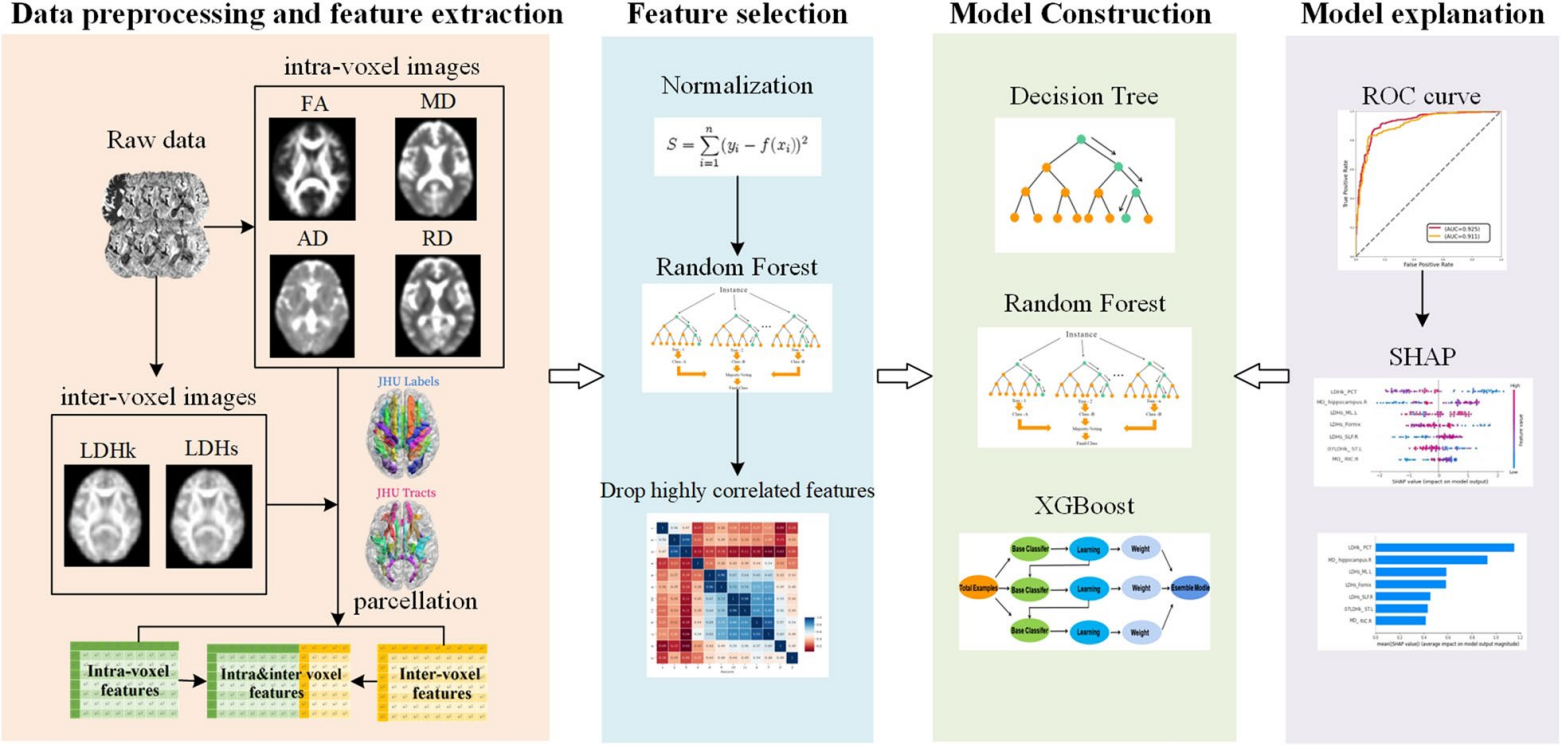
Flowchart of the study. First, a total of 420 features were extracted for each subject, and an intravoxel feature group (280 features), an intervoxel feature group (140 features) and their combination, an intra- and intervoxel feature group, were generated. After standardizing the features, the random forest algorithm and Spearman’s correlation were carried out to reduce the dimensionality of the dataset. Finally, decision tree, random forest, and extreme gradient boosting (XGBoost) were used to discriminate between PD-MCI and PD-NC subjects. SHapley Additive exPlanation (SHAP) analysis was performed to interpret the predictive model

Finally, an additional feature attribution method, SHAP, was used to characterize the optimal model and identify the top contributing DTI index for classification. SHAP analysis, a model-independent method, provides insights into the model by calculating the global influence (positive or negative, feature importance ranking) of each feature on the model prediction. The workflow of this study is presented in Fig. 1.

### Statistical analysis

All statistical analyses were performed using SPSS 22.0 software, and a two-tailed *p* < 0.05 was considered significant. The Shapiro‒Wilk test (S‒W test) was conducted to assess the normality of the distributions of continuous variables. Based on the normality of the data, the t test or the Mann‒Whitney U test was conducted to confirm the differences between groups. Measurement data with or without a normal distribution are expressed as the mean ± standard deviation (*χ* ± s) or median and interquartile M (P25-P75), respectively, while enumeration data are expressed as (*n*). Finally, partial correlation was adopted to evaluate the relationship among eigenvalues after selection and MoCA scores, MDS-UPDRS-III scores, H&Y stage, and disease duration.

## Results

### Demographic characteristics

Five patients were excluded due to a diagnosis of PDD, three patients were excluded due to structural MR abnormalities, and five patients were excluded due to the inability to cooperate with MR or clinical assessments. Finally, 120 PD patients without dementia (including 52 PD-NC patients and 68 PD-MCI patients) were included in this study. There were no significant differences in sex, age, education level, disease duration, H&Y stage, MDS-UPDRS-III, LEDD, or HAMD between the two groups. The MMSE, MoCA, CDT, AVLT, TMT-A and TMT-B scores of patients in the PD-MCI group were lower than those of patients in the PD-NC group. The demographic characteristics of all participants are detailed in Table 1.

**Table 1:** Participant demographics and clinical information

	PD-MCI (n = 68)	PD-NC (n = 52)	P value
Age (years)	64.43 ± 7.86	62.73 ± 7.79	0.242^a^
Gender (male/female)	36/32	28/24	0.922^b^
Education (years)	9.0 (7.3–12.0)	9.0 (9.0–12.0)	0.111^c^
Disease duration (years)	4.5 (2.0–7.0)	3 (2.0–6.0)	0.510^c^
MDS-UPDRS-IIII	25.55 ± 10.84	23.54 ± 13.47	0.366^a^
H&Y stage	2.0 (1.5–3.0)	2.0 (1.5–2.5)	0.337^c^
LEDD (mg)	363.97 ± 73.10	356.02 ± 108.17	0.632^a^
MMSE	25.17 ± 2.20	28.47 ± 1.33	< 0.001^a**^
MoCA	21.65 ± 2.78	26.71 ± 1.59	< 0.001^a*^
CDT	6.5(5.0–9.0)	8.0 (7.0–10.0)	< 0.001^c**^
AVLT	30.31 ± 4.44	40.48 ± 3.23	< 0.001^a**^
TMT-A	74.49 ± 18.36	67.65 ± 18.30	0.045^a**^
TMT-B	221.18 ± 59.78	167.14 ± 60.17	< 0.001^a**^
HAMD	13.08 ± 6.81	11.85 ± 6.41	0.317^a^

Data are represented as the mean ± SD or median (p25-p75) values depending on the distribution of the variables. a Two sample t test; b Chi-square test; c Mann‒Whitney U test; *P < 0.05. *PD-MCI* Parkinson’s disease with mild cognitive impairment, *PD-NC* Parkinson’s disease with normal cognition, *MDS-UPDRS-IIII* Movement Disorder Society Unified Parkinson’s Disease Rating Scale-Part III, *H&Y stage* Hoehn and Yahr stage, *LEDD* levodopa equivalent daily dose, *MMSE* Minimum Mental State Examination, *MoCA* Montreal Cognitive Assessment, *CDT* Clock Drawing Test, *AVLT* Auditory Verbal Learning Test, *TMT-A, TMT-B* Trail Making Test A and B, *HAMD* Hamilton Depression Scale

### Feature selection

**Fig. 2:**
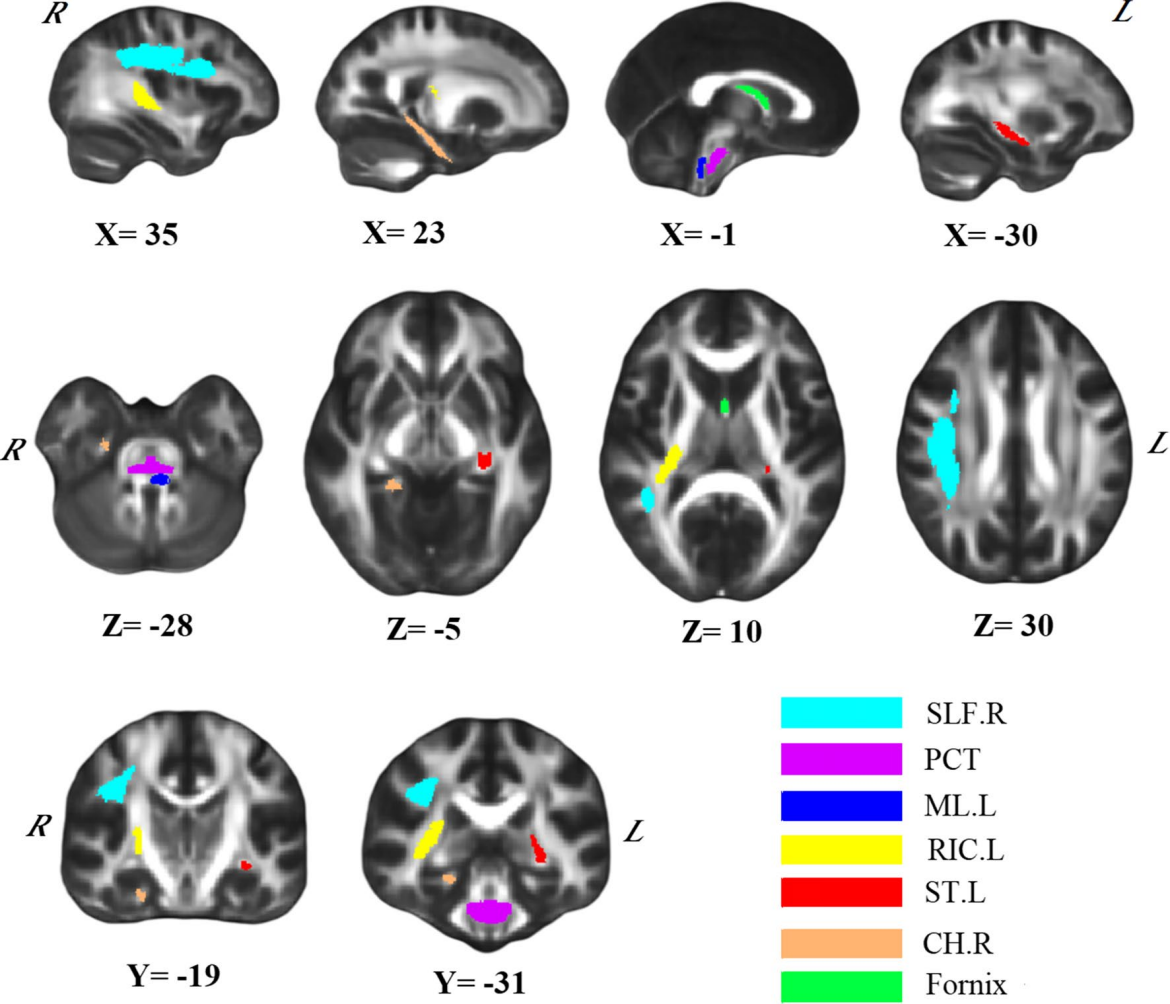
Seven features were selected for discriminating patients with PD-MCI and PD-NC. In this case, the results are displayed on a canonical FMRIB58_FA template. “.R and .L” in the text indicate the right and left sides, respectively. Neurologic conventions and MNI coordinates are used. *MD* mean diffusivity, *LDHs* local diffusion homogeneity (LDH) using Spearman’s rank correlation coefficient, *LDHk* LDH using Kendall’s coefficient concordance, *RIC* retrolenticular part of the internal capsule, *PCT* pontine crossing tract, *ML* medial lemniscus, *ST* fornix (cres)/stria terminalis, *SLF* superior longitudinal fasciculus, *CH* = cingulum (hippocampus)

For the intravoxel metrics model, 8 features were retained after RF feature selection, and 2 features were excluded after Spearman’s rank correlation analysis. For the intervoxel metrics model, 5 features were retained after RF feature selection, and no features were excluded after Spearman’s rank correlation analysis. For the combined metrics model, the RF feature selection retained the top 12 features in terms of feature importance. After Spearman’s rank correlation analysis, 5 features were excluded. Finally, the 7 most discriminative DTI features were retained (including three LDHs, two LDHk and two MD values). The WM structural connectivity areas with classification significance were mainly located in the brainstem—pontine crossing tract (PCT), medial lemniscus (ML), right cingulum (hippocampus) and left fornix (cres)/stria terminalis. Between-group comparisons showed that patients with PD-MCI exhibited greater MD in the PCT than patients in the PD-NC group. Table 2 lists the details of the feature group for the combined metrics model, and the corresponding brain region locations are shown in Fig. 2.

**Table 2:** Statistical descriptions and p values for all 7 selected features

ID	Feature type	Brain region	PD-MCI (n = 68)	PD-NC (n = 52)	P value
1	LDHk	PCT	0.807 ± 0.050	0.819 ± 0.048	0.186
2	MD	CH. R	(0.699 ± 0.066) ×10 − 3	(0.673 ± 0.064) ×10 − 3	0.035*
3	LDHs	Fornix	0.783 ± 0.118	0.801 ± 0.122	0.430
4	LDHs	ML.L	0.827 ± 0.082	0.842 ± 0.060	0.250
5	LDHs	SLF.R	0.814 ± 0.053	0.820 ± 0.058	0.603
6	LDHk	ST. L	0.717 ± 0.068	0.739 ± 0.069	0.088
7	MD	RIC.R	(0.798 ± 0.075) ×10 − 3	(0.778 ± 0.086) ×10 − 3	0.191

The ID numbering order is consistent with the SHAP value. “.R and.L” in the text indicate the right and left sides, respectively. All data are represented as the mean ± SD, and we applied two-sample t tests for comparisons between groups. *P < 0.05. *PD-MCI* Parkinson’s disease with mild cognitive impairment, *PD-NC* Parkinson’s disease with normal cognition, *LDHk* LDH using Kendall’s coefficient concordance, *LDH* LDH using Spearman’s rank correlation coefficient, *MD* mean diffusivity, *RIC* retrolenticular part of the internal capsule, *PCT* pontine crossing tract, *ML* medial lemniscus, *ST* fornix (cres)/stria terminalis, *SLF Right* superior longitudinal fasciculus, *CH* cingulum (hippocampus)

### Comparison between the models

**Fig. 3:**
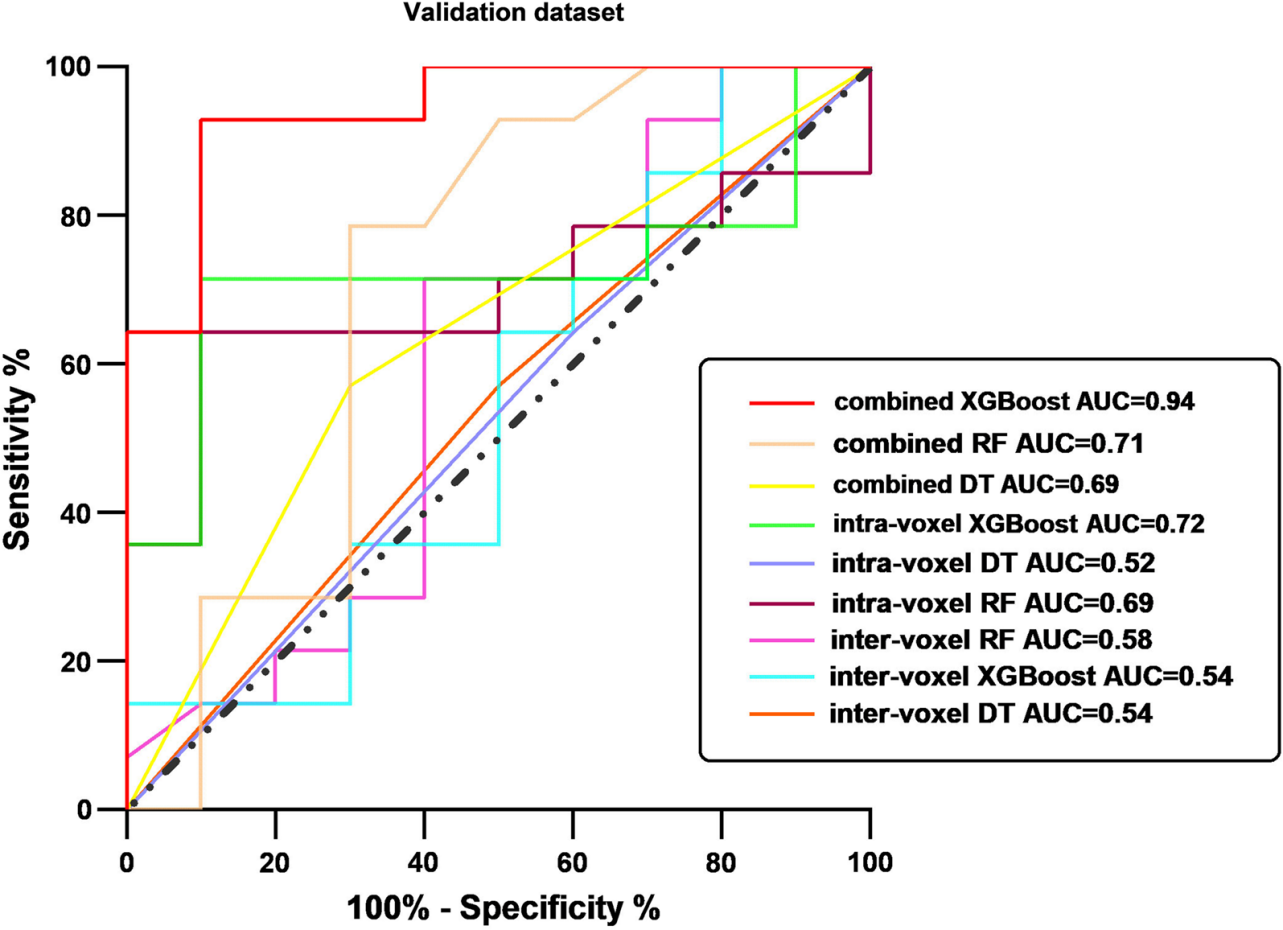
ROC curves of each model index in the test datasets. The area under the ROC curve (AUC), accuracy, sensitivity, specificity, positive predictive value (PPV), and negative predictive value (NPV) were calculated and are shown in Table 3

The evaluation scores for accuracy, sensitivity, specificity, and area under the curve (AUC) for each model are shown in Table 3; Fig. 3. The XGBoost model based on the combined intra- and intervoxel DTI indices had the highest classification performance; the test set AUC was 0.94, the accuracy was 91.67%, the sensitivity was 92.86% and the specificity was 90.00%. The AUC difference between combined_XGBoost and intravoxel_XGBoost did not reach statistical significance (P = 0.07, Delong test), and the AUC of combined_XGBoost was significantly higher than that of all other models except intravoxel_XGBoost (P < 0.01, Delong test). In addition, the intravoxel_RF model had the highest specificity (90.00%) and moderate sensitivity (64.29), and the intervoxel_XGBoost model had the highest sensitivity (100.00%) and very low specificity (10.00%).

**Table 3:** Classification performance of the different models

Models	Training dataset (n = 96)						Test dataset (n = 24)					
	Accuracy (%)	Sensitivity (%)	Specificity (%)	PPV (%)	NPV (%)	AUC	Accuracy (%)	Sensitivity (%)	Specificity (%)	PPV (%)	NPV (%)	AUC
Intra-voxel												
DT	100.00	100.00	100.00	100.00	100.00	1.00	54.17	64.29	40.00	60.00	44.44	0.52
RF	100.00	100.00	100.00	100.00	100.00	1.00	75.00	64.29	90.00	90.00	64.29	0.69
XGBoost	100.00	100.00	100.00	100.00	100.00	1.00	75.00	71.43	80.00	83.33	66.67	0.72
Inter-voxel												
DT	100.00	100.00	100.00	100.00	100.00	1.00	54.17	57.14	50.00	61.54	45.45	0.54
RF	100.00	100.00	100.00	100.00	100.00	1.00	66.67	71.43	60.00	71.43	60.00	0.58
XGBOOST	100.00	100.00	100.00	100.00	100.00	1.00	62.50	100.00	10.00	60.87	100.00	0.54
Combined intra- and intervoxel												
DT	100.00	100.00	100.00	100.00	100.00	1.00	66.67	57.14	80.00	80.00	57.14	0.69
RF	100.00	100.00	100.00	100.00	100.00	1.00	75.00	78.57	70.00	78.57	70.00	0.71
XGBoost	100.00	100.00	100.00	100.00	100.00	1.00	91.67	92.86	90.00	92.86	90.00	0.94

*DT* decision tree, *RF* random forest, *XGBoost* eXtreme Gradient Boosting, *PPV *positive predictive value, *NPV* negative predictive value, *AUC* area under the receiver operating characteristic curve

### Feature importance

The SHAP summary plot of each prediction in the combined XGBoost model is presented in Additional file 2:  Fig. S1. According to the SHAP value, the LDHk of PCT served as the most important feature. The MD of the right cingulum (hippocampus) and the LDHs of the right ML were also important for model prediction (Additional file 3: Fig. S2).

### Correlation analysis

The correlations of each DTI metric value with MoCA score, MDS-UPDRS-III score, H & Y stage, and disease duration are summarized in Additional file 5:  Table S2. After controlling for disease duration and MDS-UPDRS-III score, the LDHk values in the PCT were positively correlated with the MoCA scores (r = 0.246, P = 0.007), the MD values in the right cingulum (hippocampus) were negatively correlated with the MoCA scores (r = −0.206, P < 0.001), and the LDHk values in the left fornix (cres)/stria terminalis were positively correlated with the MoCA scores (r = 0.223, P = 0.015).

## Discussion

This study succeeded in developing a machine learning model based on DTI metrics to accurately discriminate PD-MCI patients from PD patients without dementia. The main contributions were as follows: First, to our knowledge, this was the first time that LDHs and LDHk (novel intervoxel DTI indices) were used as classification metrics. We confirmed that these metrics can be used as a complement to intravoxel metrics to improve the classification accuracy for PD-NC and PD-MCI patients. Second, the XGBoost model based on the combined intra- and intervoxel metrics achieved a classification accuracy of 91.67% and an AUC of 0.94 in the test dataset and was the best performing model. Finally, by applying the SHAP method to interpret the best model, the LDHk values of the pontine crossing tract (PCT) were found to be important features, as were the MD values of the right cingulum (hippocampus).

Although neuropsychological testing remains the primary method for assessing the presence or absence of cognitive decline in PD patients, neuroimaging studies have observed brain structural and functional changes in patients with PD-MCI by measuring grey matter volume, white matter damage, and resting-state functional activity [27]. For years, machine learning studies used neuroimaging or electrophysiology data to build classifiers for PD-MCI. One study found that electroencephalogram (EEG) signals achieved 84% classification accuracy in identifying PD-MCI patients [28]. Zhang et al.‘s study [29] combined EEG and grey matter structural MRI as input features to identify patients with PD-MCI, achieving the highest accuracy of 77%. Moreover, Lenfeldt et al. found that white matter DTI values are more sensitive neuroimaging indicators than grey matter atrophy [11]. To the best of our knowledge, previous DTI studies have mainly focused on the differences in intravoxel diffusion parameters, finding that cognitive impairment is associated with decreased FA and increased MD, AD, and RD values in multiple WM regions, particularly in the predominantly anterior WM tracts [12, 30]. Haller et al. attempted to use machine learning algorithms based on intravoxel DTI indicators to diagnose Parkinson’s disease [31], with accuracies of up to 97%. However, we have not found any research utilizing machine learning models with DTI features to automatically identify PD-MCI patients. Our study confirms that intervoxel metrics that reflect the microstructural consistency of white matter fibre tracts can complement traditional intravoxel metrics to reveal a comprehensive picture of WM alterations. The reason this phenomenon has not been described appears to be that previous works did not consider intervoxel DTI metrics. To our knowledge, this is the first study to automatically identify PD-MCI using machine learning methods based on both intra- and intervoxel DTI features. Regarding the machine learning-based algorithms, we found that XGBoost-based algorithms achieved better performance, which is consistent with previous research. Lee et al. confirmed that an XGBoost model based on electroencephalography signals had a good effect in the diagnosis of PD [21], with the highest accuracy rate of 71.4%. Shibata et al. applied the XGBoost model based on quantitative susceptibility mapping (a type of MR method reflecting iron deposition) features to classify PD-MCI and PD-NC patients, achieving an accuracy of 79.1% [32].

This study used SHAP analysis to interpret the best model, which revealed that the MD values of the right cingulum and the LDHk values of the PCT were the most important features, as well as the LDH values of the ML, another region of the brainstem. Statistical analysis revealed that the LDHk value of the PCT in the PD-MCI group was lower than that in the PD-NC group and that in general, the LDHk value of the PCT was positively correlated with the MoCA score. One of the pathological hallmarks of PD is dopaminergic neuron loss, and postmortem studies have confirmed that human brainstem regions, such as the substantia nigra, red nucleus, medial lemniscus, and pontine nucleus, highly express D2 dopamine receptor mRNAs [33]. Furthermore, the PCT and ML are the main structural connections of the cerebello-thalamo-cortical (CTC) circuits. Various lines of evidence suggest that the CTC circuits play a critical role in the cognitive symptoms of PD. Pathological studies have confirmed the presence of landmark Lewy body pathology aggregates in the cerebellar nuclei and adjacent white matter displayed in PD patients [34, 35]. Neuroimaging studies have confirmed that the CTC loop mediates the involvement of the cerebellum in higher-order cognitive processes, such as planning, verbal fluency, mental flexibility, abstract reasoning, and working memory; its dysfunction contributes to cognitive dysfunction in PD [36, 37]. Therefore, our results further support the notion that the CTC circuitry is affected by disease-specific impairments in PD and contributes to cognitive dysfunction in PD. Moreover, the PCT and ML contain topologically arranged projection fibres, and adjacent voxels may project to very different neocortices. Thus, it is possible that when one of the voxels suggests damage, its neighbours remain normal. LDH estimates the overall consistency of diffusion of water molecules between a voxel and its neighbours, and so abnormalities in the PCT and ML may be more sensitive to LDH.

In addition, the MD value of the right cingulum (hippocampus) was the second most important feature and was significantly negatively correlated with MoCA scores. Several studies have shown susceptibility alterations in the hippocampus in patients with PD-MCI. The hippocampus plays an important role in the interaction between dopamine transmission and hippocampal synaptic remodelling, and an imbalance in this interaction leads to dementia [38]. Neuropathological studies have observed Lewy body pathology (accumulations of the protein alpha-synuclein) in the hippocampus of PD patients, and the degree of cognitive impairment is correlated with the degree of Lewy body deposition in the hippocampus [39]. Increased MD indicates extensive cellular damage, including oedema and necrosis [10]. Multimodal MRI studies have confirmed that injury to the structural integrity and connectivity of the fornix-hippocampal projections is associated with decreased memory test scores in PD patients [40]. DTI studies have confirmed that PD-NC patients showed increased fornix MD values compared with those of HCs [40], and PDD patients showed lower hippocampal FA values than PD patients without dementia [13]. In this study, the LDHk value of the left fornix (cres)/stria terminalis was also decreased in the PD-MCI group. This finding indicates that microstructural damage to the fornix-hippocampal projection plays an important role in the cognitive impairment in PD. The pathological changes in the hippocampus may be relatively dispersed, and the transition from the normal area to the abnormal area is not as sudden as that in the PCT and ML. Therefore, the MD index of the hippocampus has classification importance.

Several limitations of our study should be noted. First, this work is a retrospective study, and prospective studies are needed in the future to validate whether the proposed method can predict the conversion of PD-NC to PD-MCI. Additionally, although this study adopted the simpler level I diagnostic criteria for PD-MCI, one study confirmed that level II criteria did not add value to the level I criteria [24]. Second, this study did not further subdivide patients according to cognitive impairment, specifically including (1) frontal-dominant impairment and (2) posterior-cortical-dominant impairment, which is a future research direction we plan to pursue. Finally, the present study only explored the predictive ability of DTI parameters in white matter brain regions for PD-MCI patients, which may be a rather one-sided analysis, and multimodal data (including spatial and temporal features) are needed in the future to fully explore the mechanisms of PD-MCI and increase accuracy of machine learning models, as studied by Bianchetti et al [41].

## Conclusion

In conclusion, a machine learning model trained with DTI metrics extracted from atlas-based WM segmentation shows potential in differentiating individuals with PD-MCI from PD patients without dementia. Specifically, the combined application of intra- and intervoxel diffusion measures can provide more comprehensive information about white matter alterations and improve classification accuracy. XGBoost models based on combined DTI indices are particularly promising classifiers with high classification accuracy. After further validation, the model may become a valuable tool in supporting PD-MCI clinical diagnostic systems.

## Supplementary Information

**Additional file 1:** MRI data acquisition and preprocessing.

**Additional file 2:** **Figure S1.** SHapley Additive exPlanation. SHAP summary plot showing the values of features in every sample. Each line represents a feature, and the abscissa represents the SHAP value. Each dot represents a sample. Feature Importance: The mean absolute SHAP value of each feature.

**Additional file 3:** **Figure S2.** Overview of correlations of the regional mean DTI values with MoCA scores. “.R” and “-.L” indicate the right and left sides, respectively. Abbreviations: PD-MCI= Parkinson's disease with mild cognitive impairment; PD-CN = Parkinson's disease with normal cognition; MoCA=Montreal CognitiveAssessment; CH=cingulum; ST=fornix/stria terminalis; MD=mean diffusivity; LDHk=local diffusion homogeneity using Kendall's coefficient concordance.

**Additional file 4:** **Table S1.** Hyperparameters of different models for classifying PD-MCI vs. PD-NC.

**Additional file 5:** **Table S2.** All correlations between the DTI values and clinical scores among all participants.

## Data Availability

The datasets from the current study are available from the corresponding author upon reasonable request.

## Abbreviations

AUC: Area under the curve
DTI: Diffusion tensor imaging
FA: Fractional anisotropy
RF: Random forest
LDHk: Local diffusion homogeneity using Kendall’s coefficient concordance
LDHs: Local diffusion homogeneity using Spearman’s rank correlation coefficient
MD: Mean diffusivity
MDS-UPDRS-III: Movement Disorder Society Unified Parkinson’s Disease Rating Scale Part III
MoCA: Montreal Cognitive Assessment
PCT: pontine crossing tract
PD: Parkinson’s disease
PD-MCI: Parkinson’s disease with mild cognitive impairment
PD-NC: Parkinson’s disease with normal cognition
ROC: Receiver operating characteristic
WM: White matter
XGBoost: Extreme gradient boosting
