# Supplementary material for: Detection of mild cognitive impairment in Parkinson’s disease using gradient boosting decision tree models based on multilevel DTI indices
Source: J Transl Med. 2023 May 8;21:310. doi: 10.1186/s12967-023-04158-8 (PMC10165759; doi:10.1186/s12967-023-04158-8)
Supplement: Supplementary file 1 — Additional file 1: MRI data acquisition and preprocessing. [file 12967_2023_4158_MOESM1_ESM.docx]

MRI data acquisition and preprocessing

1. DTI data acquisition

All participants were examined on a Magnetom Verio 3.0 T MRI scanner (Siemens Medical Solutions, Erlangen, Germany) with a 32-channel head coil. Diffusion weighted images were acquired in parallel with the anterior-posterior commissural plane using an echo planar imaging sequence. The scanning parameters were as follows: repetition time (TR)/ echo time (TE) =10300/95 ms, field of view (FOV) = 256x256 mm^2^, matrix = 128x128, voxel size = 2.0x2.0x2.0mm^3^, slice thickness = 2mm, number of directions = 64, b value = 1000 sec/mm2.

2. DTI data preprocessing

We used FSL 5.0.9(https://fsl.fmrib.ox.ac.uk/) and PANDA (Pipeline for Analyzing braiN Diffusion imAges, http://www.nitrc.org/projects/panda/) to conform the pre-processing and atlas-based analysis (ABA). The pre-processing steps included conversion to DICOM-to-NIFTI format, manual quality assessment to check for missing slices, visual assessment to identify intensity artifacts, and brain extraction using FSL’s ‘BET’ [1]. Eddy current and motion correction was performed using FSL’s ‘eddy correct’ tool that employs an affine transformation between the baseline b = 0 image and the gradient images. Based on the rotation parameter in the affine transformation, the gradients were rotated using ‘fdt_rotate_bvecs’ to match the transformed images. To reduce the Rician noise in the DWI images, a joint linear minimum mean square error (jLMMSE) filter with default estimation and filtering radius was employed. The diffusion tensor images were then reconstructed using least square approximations and manually checked to ensure that the tensors aligned with the underlying anatomy and no gradient flip were required.

**3 Feature Extraction**

In this study, we calculated six types of DTI indicators. Four commonly evaluated intra-voxel diffusivity metrics, FA(a normalized SD of the eigenvalues), MD(a direction-averaged measure), AD(apparent diffusivity parallel to the underlying tissue tract), and RD(apparent diffusivity perpendicular to the underlying tissue tract), were obtained from the tensor matrix. In addition, we calculated an inter-voxel diffusivity metric called local diffusion homogeneity(LDH) using Spearman's rank correlation coefficient(LDH) and Kendall's coefficient concordance(LDHk); the specific calculation method was performed according to a previous study[2]. The atlas-based analysis(ABA) method in the PANDA software package was selected for feature extraction. According to the "John Hopkins University ICBM-DTI-81 White Matter Labels" and "John Hopkins University White Matter Tractography(http://cmrm.med.jhmi.edu)" atlases[3], the whole-brain WM was divided into 70 regions of interest, and the mean DTI parameters were extracted for each region. Ultimately, 280 intra-voxel and 140 inter-voxel features were extracted for each subject.

1. Popescu, V., et al., *Optimizing parameter choice for FSL-Brain Extraction Tool (BET) on 3D T1 images in multiple sclerosis.* Neuroimage, 2012. **61**(4): p. 1484-1494.

2. Gong, G., *Local diffusion homogeneity (LDH): an inter-voxel diffusion MRI metric for assessing inter-subject white matter variability.* PLoS One, 2013. **8**(6): p. e66366.

3. Hua, K., et al., *Tract probability maps in stereotaxic spaces: analyses of white matter anatomy and tract-specific quantification.* Neuroimage, 2008. **39**(1): p. 336-347.
