# Supplementary figures and images for: Detection of mild cognitive impairment in Parkinson’s disease using gradient boosting decision tree models based on multilevel DTI indices
Source: J Transl Med. 2023 May 8;21:310. doi: 10.1186/s12967-023-04158-8 (PMC10165759; doi:10.1186/s12967-023-04158-8)

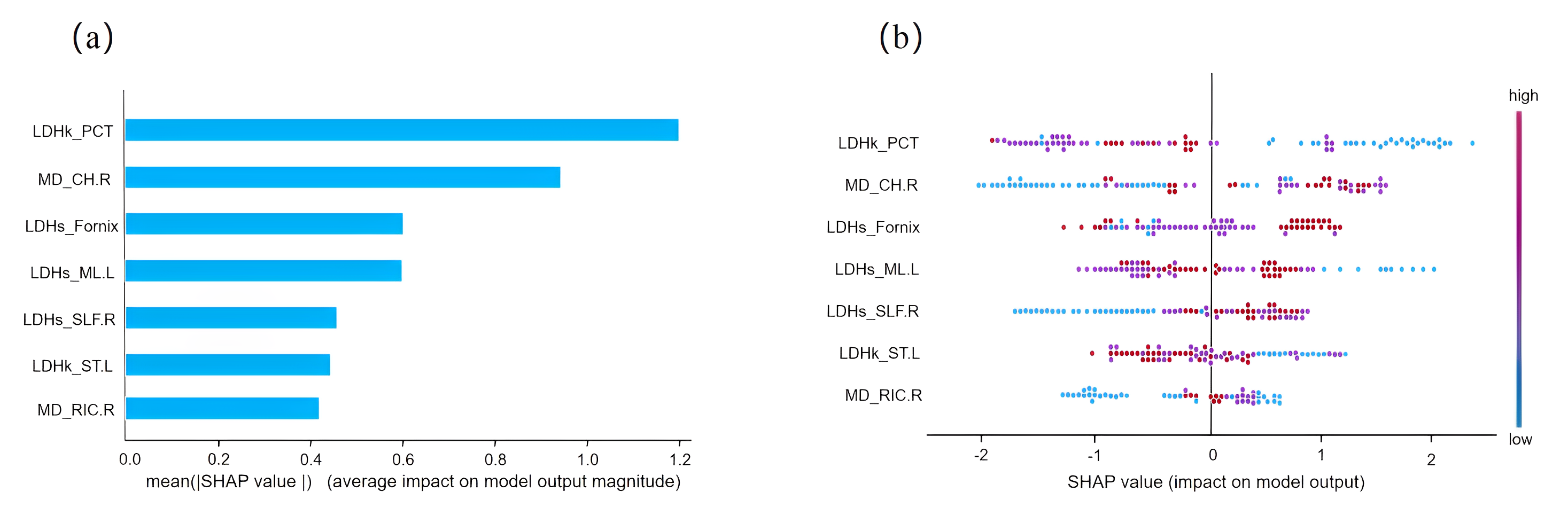

Supplement: Supplementary file 2 — Additional file 2: Figure S1. SHapley Additive exPlanation. SHAP summary plot showing the values of features in every sample. Each line represents a feature, and the abscissa represents the SHAP value. Each dot represents a sample. Feature Importance: The mean absolute SHAP value of each feature. [file 12967_2023_4158_MOESM2_ESM.png]

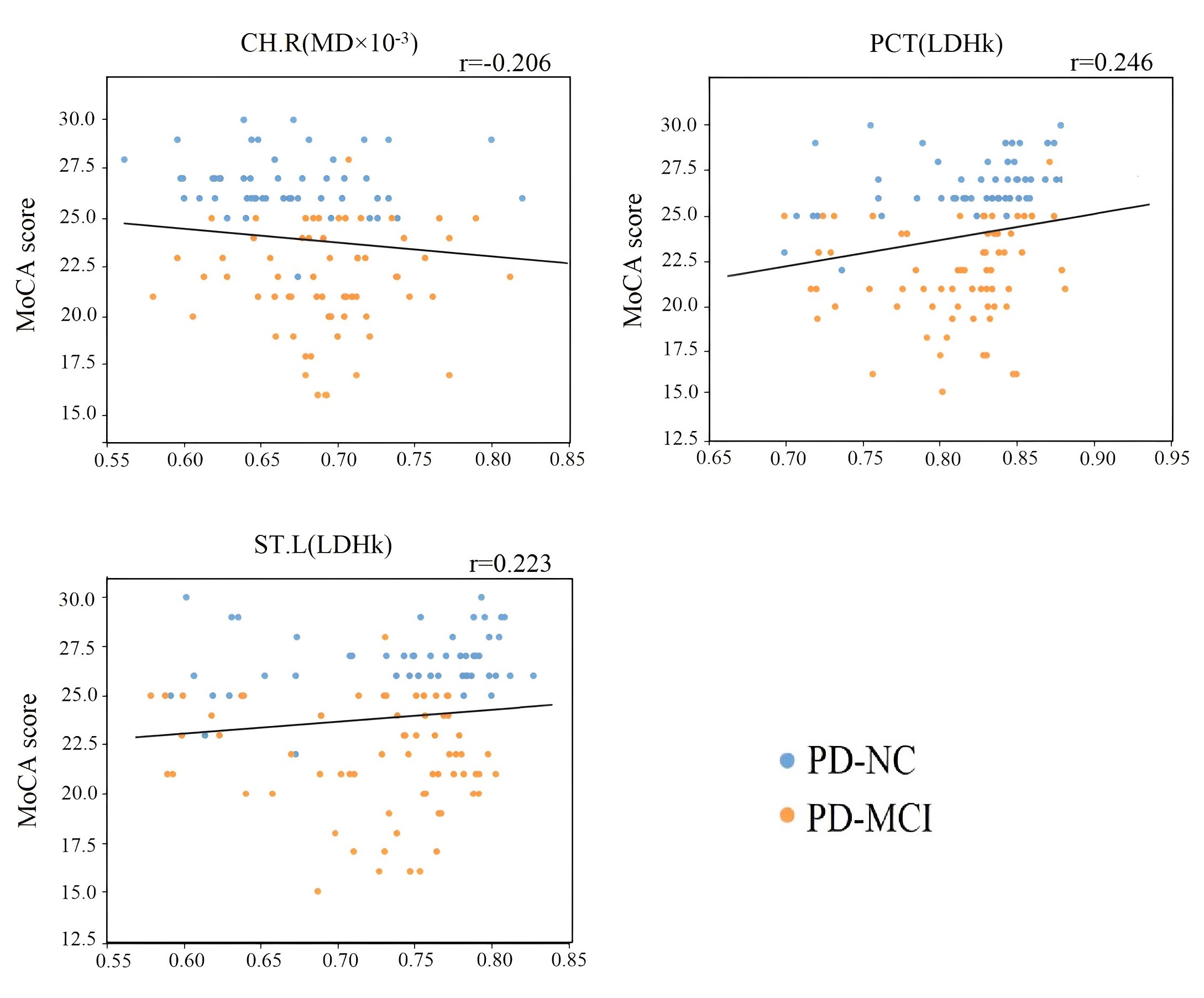

Supplement: Supplementary file 3 — Additional file 3: Figure S2. Overview of correlations of the regional mean DTI values with MoCA scores. “.R” and “-.L” indicate the right and left sides, respectively. Abbreviations: PD-MCI= Parkinson's disease with mild cognitive impairment; PD-CN = Parkinson's disease with normal cognition; MoCA=Montreal CognitiveAssessment; CH=cingulum; ST=fornix/stria terminalis; MD=mean diffusivity; LDHk=local diffusion homogeneity using Kendall's coefficient concordance. [file 12967_2023_4158_MOESM3_ESM.jpg]
