# Supplementary material for: Detection of mild cognitive impairment in Parkinson’s disease using gradient boosting decision tree models based on multilevel DTI indices
Source: J Transl Med. 2023 May 8;21:310. doi: 10.1186/s12967-023-04158-8 (PMC10165759; doi:10.1186/s12967-023-04158-8)
Supplement: Supplementary file 4 — Additional file 4: Table S1. Hyperparameters of different models for classifying PD-MCI vs. PD-NC. [file 12967_2023_4158_MOESM4_ESM.docx]

Table S1 Hyperparameters of different models for classifying PD-MCI vs. PD-NC.

| Model | parameters |
| --- | --- |
| Decision Trees | criterion=Gini Impurity, max_depth=1, max_leaf_nodes=2, min_impurity_decrease=0,  min_samples_split=2, min_samples_leaf=1, min_samples_split=2 |
| Random Forest | n estimators=10, criterion= entropy, max_depth=1, min_samples_split=2,  min_samples_leaf=1, max_features=1, max_leaf_nodes=1, min_impurity_decrease=0 |
| XGBoost | n_estimators, booster=gbtree, subsample=0.8, max_depth=3, gamma=0.1, min_child_weigh=1, max_leaf_nodes=1, reg_lambda=1, reg_lambda=0 |

Abbreviation: XGBoost=eXtreme Gradient Boosting
