# Supplementary material for: Detection of mild cognitive impairment in Parkinson’s disease using gradient boosting decision tree models based on multilevel DTI indices
Source: J Transl Med. 2023 May 8;21:310. doi: 10.1186/s12967-023-04158-8 (PMC10165759; doi:10.1186/s12967-023-04158-8)
Supplement: Supplementary file 5 — Additional file 5: Table S2. All correlations between the DTI values and clinical scores among all participants. [file 12967_2023_4158_MOESM5_ESM.docx]

**Table S2 All correlations between the DTI values and clinical scores among all participants**

| ROIs | MoCA | MDS−UPDRS III | H&Y stages | Disease duration |
| --- | --- | --- | --- | --- |
| PCT (LDHk) | 0.206* | 0.348* | 0.120 | 0.183* |
| CH (MD) | -0.135 | -0.010 | 0.045 | -0.149 |
| ML. Left (LDHs) | 0.124 | 0.313* | 0.091 | 0.059 |
| ST. Left (LDHk) | 0.122 | 0.403* | 0.094 | 0.178 |
| Fornix (LDHs) | 0.094 | 0.306* | 0.036 | 0.212* |
| RIC. Right (MD) | -0.021 | -0.215 | -0.057 | -0.154 |
| SLF. Right (LDHs) | 0.115 | 0.423* | 0.158 | 0.253* |

Note. - H&Y stages and disease duration assessed using Spearman’s correlation; *Indicates P<0.05

Abbreviations: ROIs = region of interests; MoCA = Montreal Cognitive Assessment; MDS−UPDRS III = Movement Disorder Society Unified Parkinson's Disease Rating Scale Part III score; H&Y stages=Hoehn and Yahr stages; PCT=pontine crossing tract; ML=medial lemniscus; CH=cingulum (hippocampus); ST=fornix (cres)/stria terminalis; RIC=retrolenticular part of the internal capsule; SLF= superior longitudinal fasciculus; LDHk=LDH metric Kendall’s coefficient concordance; LDH=LDH metric uses Spearman's rank correlation coefficient; MD=mean diffusivity.
